# Immune Microenvironment in Glioblastoma Subtypes

**DOI:** 10.3389/fimmu.2018.01004

**Published:** 2018-05-08

**Authors:** Zhihong Chen, Dolores Hambardzumyan

**Affiliations:** Department of Pediatrics, Aflac Cancer and Blood Disorders Center, Children’s Healthcare of Atlanta, Emory University School of Medicine, Atlanta, GA, United States

**Keywords:** glioblastoma, macrophages, microglia, immunotherapy, tumor-associated macrophages, tumor microenvironment

## Abstract

Glioblastomas (GBMs) are the most common and aggressive primary brain tumors. Due to their malignant growth and invasion into the brain parenchyma coupled with resistance to therapy, GBMs are among the deadliest of all cancers. GBMs are highly heterogeneous at both the molecular and histological levels. Hallmark histological structures include pseudopalisading necrosis and microvascular proliferation. In addition to high levels of intratumoral heterogeneity, GBMs also exhibit high levels of inter-tumoral heterogeneity. The major non-neoplastic cell population in the GBM microenvironment includes cells of the innate immune system called tumor-associated macrophages (TAMs). Correlative data from the literature suggest that molecularly distinct GBM subtypes exhibit differences in their microenvironment. Data from mouse models of GBM suggest that genetic driver mutations can create unique microenvironments. Here, we review the origin, features, and functions of TAMs in distinct GBM subtypes. We also discuss their interactions with other immune cell constituents and discuss prospects of therapeutically targeting TAMs to increase the efficacy of T-cell functions.

## Introduction

Glioblastomas (GBMs) are the most common and aggressive malignant primary brain tumors in adults (1). GBM cells are characterized by diffuse infiltration of the adjacent brain parenchyma and the development of resistance to standard treatment (2–4). The standard of care consists of surgical resection followed by radiotherapy (RT) and concomitant and adjuvant temozolomide [(TMZ); TMZ/RT→TMZ]. Despite this aggressive treatment regimen, the median survival is only around 15 months, and the 2-year survival rate is only 26.5% (5).

Glioblastomas were traditionally considered to be a single histological entity by the World Health Organization. However, a more recent characterization of the genome, epigenome, and transcriptome of GBMs has provided a higher-resolution picture of frequent alterations, based on which robust gene expression-based subtypes named proneural (PN), mesenchymal (MES), and classical (CL) were established (6–10). These analyses associated aberrations in the gene expression of platelet-derived growth factor receptor alpha (PDGFRA), neurofibromatosis type I (NF1), and epidermal growth factor receptor (EGFR) with the PN, MES, and CL subtypes, respectively. Although it is important to emphasize that multiple subtypes could co-exist within a single tumor both at the regional and at the single-cell levels (11, 12), the designated subtypes reflect the dominant transcriptional program of a specific tumor within a particular time and space of sample isolation (10). By analyzing copy number alterations from The Cancer Genome Atlas (TCGA) data to evaluate the presence of NF1 loss, PDGFRA amplification, and EGFR amplification in human GBM (hGBM) samples when co-incidence of mutations was excluded, we demonstrated that NF1 loss, PDGFRA amplification, and EGFR amplification tend to occur most frequently in MES, PN, and CL hGBMs, respectively (13, 14).

As described above, GBMs display a high degree of inter- and intratumor heterogeneity. The tumor microenvironment, in which these tumor cells develop and grow, further adds to this diversity. The GBM microenvironment contains an array of non-neoplastic cells, including infiltrating and resident immune cells, vascular cells, and other glial cells. Particular emphasis has been placed on various non-neoplastic constituents of the immune system, especially tumor-associated macrophages (TAMs). TAMs are the dominant infiltrating immune cell population, constituting ~30–40% of the cells in a GBM (15, 16). These cells have been shown to engage in reciprocal interactions with neoplastic tumor cells to promote tumor growth and progression (17–20). With the advent of immunotherapeutic strategies for GBM, T cells have also been the subject of increasing scrutiny (21, 22). These innate and adaptive immune cells together form the basis of our host defense, where they perform cancer immune surveillance at early stages of premalignant lesions. However, if and when the immune system is overpowered by tumor burden during cancer development, cancers can escape this surveillance and become uncontrollable. In doing so, cancers also recruit these immune cells and methodically turn them into their accomplices (23), effectively converting the immune system from protective to detrimental. The task we are facing now as immuno-neuro-oncologists is to re-educate and re-invigorate these immune cells and to rectify their actions to be once again advantageous. This review aims to analyze the most recent findings and to assess whether genetic driver mutations can determine the expression profile of non-neoplastic cells and/or can play an important role in predicting tumor response to immunotherapy. Our goal is to promote discussion with regard to subtype-oriented immunotherapies and to advocate for such considerations.

## Immune Composition of GBM Subtypes

Since the contribution of TAMs to tumor development is substantial, several studies utilizing gene expression data from the TCGA and the Gene Expression Omnibus databases have demonstrated an enrichment in immune response-related gene expression, especially of TAM genes, in the MES subtype of GBM compared to the other subtypes (15), suggesting that TAMs could play a subtype-specific role in GBM. Despite extensive correlative studies and *in vitro* experiments implying that TAMs may play differential roles in GBM subtypes, to date, there are still no systemic functional studies corroborating this hypothesis. On the contrary, despite emerging evidence from both mouse models and TCGA analysis of hGBM (10) showing that NF1 deficiency results in an increased TAM infiltration, the clinical significance of this finding is not apparent. Clinically, the subtypes have not been established as predictive biomarkers for survival (8), although accumulating preclinical evidence has indicated that subtype-specific treatment may preferentially benefit patients. It is still not understood, however, what controls the differences in immune composition in GBM subtypes. One scenario is that tumor-associated or tumor-specific antigens, driven by genetic mutations, are differentially presented in different subtypes, which shapes the various molecular immune responses and results in the observed differential accumulation of immune cells (8, 24).

Glioblastoma creates a proangiogenic and inflamed microenvironment, which leads to an increased expression of adhesion molecules on the endothelial cells and reduced tight junctions, thereby a highly permeable blood–brain barrier (BBB). These changes support the leukocytes to exit from the blood flow by extravasating through the brain endothelial wall and infiltrate the tumor mass. Besides TAMs, many other immune cells are also found in the GBM parenchyma, although at a much lower incidence. T cells probably account for most of the lymphoid cells in GBMs; however, they represent less than 0.25% of total tumor cells isolated from hGBM biopsy samples as examined by flow cytometry (25). CD8^+^ cytotoxic T cells are cellular immune effectors that are essential for killing tumor cells, but they are only sparsely distributed in the GBM parenchyma, accounting for less than a quarter of the total CD3^+^ T cells (25). These T cells derived from GBM patients are less responsive to direct anti-CD3 stimulation *in vitro* when compared to cells obtained from healthy controls, indicating an immunosuppressed status (25). In support of this notion, it was recently shown that GBM-infiltrating T cells increased their expression of indoleamine 2,3-dioxygenase 1 (IDO1), which is an immune-inhibitory receptor and that this heightened expression correlates with poor prognosis (26). A phase I clinical trial examining the safety and utility of an IDO1 inhibitor in conjunction with TMZ in pediatric primary malignant brain tumors is currently underway (clinicaltrials.gov NCT02502708). Regulatory T cells (T_regs_) are also found in the GBM parenchyma. These cells perform immunosuppressive functions and are thought to suppress antitumor immunity in various solid tumors such as ovarian, breast, and pancreatic cancers (27). In GBM tumor cells, secreted soluble factors including CCL22 can facilitate the recruitment and retention of T_regs_ in the tumor microenvironment (28), and the amount of T_regs_ demonstrated an inverse correlation with patient survival, although it was not statistically significant (29). T_reg_ ablation eradicates T-cell-proliferative defects, restoring the functions of T cells from GBM patients *in vitro* at levels equivalent to those of healthy controls (30). Therefore, targeting T_regs_ can potentially revert tumor immune evasion, thereby facilitating tumor immunotherapy or conventional therapy.

*In silico* estimation of 22 immune cell types in human PN, CL, and MES samples has shown that there is a collective increase in several cell types in MES tumors compared to that in non-MES tumors, including CD4^+^ memory T cells, type-2 polarized macrophages, and neutrophils (10). It has been speculated that a higher level of TAMs may discourage the infiltration of effector T cells due to TAM immunosuppressive functions. However, the reasons for this hand-in-hand infiltration between TAMs and T cells in a subtype-specific manner are not evident. This could be because the T cells follow the TAMs to passively egress the bloodstream when the BBB is compromised during GBM development. However, this is unlikely in that the ratio of T cells to TAMs in the tumor is different than that in the blood, where lymphocytes considerably outnumber monocytes (progenitors of tumor TAMs). One possible explanation could be that there is a parallel increase in CCL chemokines (attracting monocyte) and CXCL chemokines (attracting lymphoid cells) in MES tumors that attract TAMs and T cells, respectively, when compared with other GBM subtypes.

Genetically engineered mouse models (GEMMs) that faithfully recapitulate hGBM subtypes are invaluable tools for enabling the investigation of subtype-specific immunopathology and for the design of relevant and effective therapies (14, 31). These specific GEMMs provide an unprecedented opportunity to define the immune cells and molecular signals that contribute to gliomagenesis and continued growth facilitated by subtype-specific glioma microenvironments. For specific questions regarding tumor–microenvironment interactions, GEMMs for various GBM subtypes represent better choice compared to other models, such as orthotopic murine allografts utilizing established murine GBM cell lines, cultured in serum for years, or hGBM xenografts, where there are well-known species incompatibilities, particularly for chemokines and their receptors. Among all of the desirable properties of these models is that they utilize immunocompetent mice, in which the immune cells and tumor cells are of the same species, eliminating species incompatibilities between chemokines, cytokines, and their respective receptors that are important for the recruitment and also the activation of various immune cell types. GEMM models of GBM will allow us to answer important biological questions regarding the relevance of differential immune infiltration in various hGBM subtypes.

## TAMs: The Origin Matters

**Figure 1:**
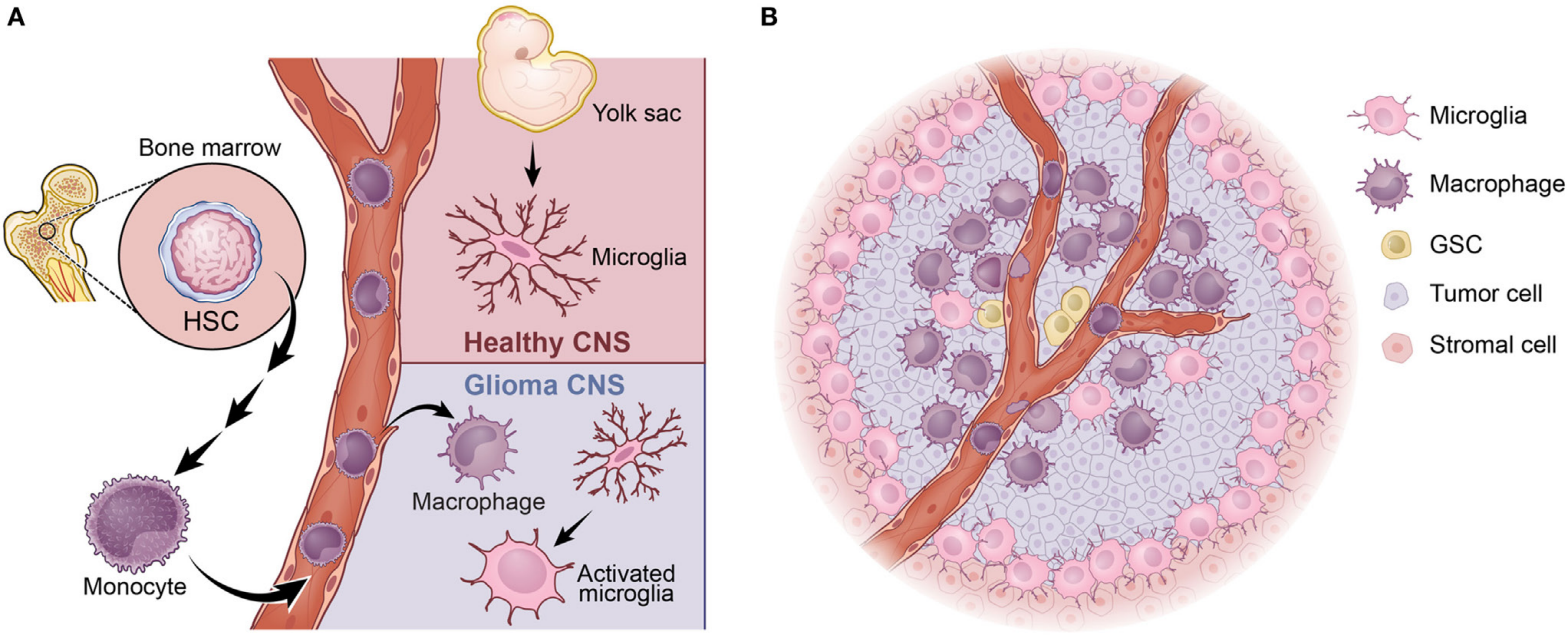
Tumor-associated macrophages (TAMs) in glioblastoma (GBM). **(A)** TAMs arise from two distinct sources: bone marrow-derived monocytes or brain-resident microglia. **(B)** In proneural GBM, the majority of TAMs are BMDMs, which largely localize in the perivascular niche, where the glioma stem-like cells (GSCs) reside. The majority of microglia are found at the peritumoral region.

Tumor-associated macrophages originate from two independent sources: brain-resident microglia and/or bone marrow-derived monocytes (Figure 1A). Microglia is the unique resident macrophages of the central nervous system (CNS) (32). Fate-mapping and lineage-tracing studies have identified immature yolk sac runt-related transcription factor 1 (Runx1)-positive progenitors as the predominant source of brain microglia. Between embryonic days 8.5 (E8.5) and E9.5, these progenitors migrate from the yolk sac into the primitive brain, where they serve as cells of origin for microglia (33). Several additional studies have subsequently revealed in mice that myeloid progenitors from the blood do not significantly contribute to the pool of adult microglia after birth. Thus, the majority of adult microglia are yolk sac-derived and are maintained by virtue of their longevity and limited self-renewal (33–36). Tracing the life span of microglia by long-term *in vivo* single-cell imaging in mice, it has been shown that neocortical resident microglia can live for about 15 months on average, almost rivaling the life span of post-mitotic neurons (37). While the naïve CNS parenchyma is occupied exclusively by resident microglia, the tumor-bearing CNS is vastly different. In the tumor-bearing brain, the BBB is impaired, and the expression of the monocyte chemoattractant family of proteins (MCPs) is increased. This results in infiltration of monocytes into tumors from the periphery, where they differentiate into macrophages. Monocytes are derived from progeny called macrophage–DC precursors, which originate from hematopoietic stem cells. These precursors differentiate into monocytes within the bone marrow and are subsequently released into the blood circulation to colonize peripheral organs (38). Mouse monocytes can be further subdivided into two main populations: Ly6C^+^, CX3CR1^int^, and CCR2^+^ inflammatory monocytes; and Ly6C^−^, CX3CR1^hi^, and CCR2^−^ circulating monocytes (39, 40). It is well established that the Ly6C^+^, CX3CR1^int^, and CCR2^+^ inflammatory monocytes leave the blood circulation and extravasate to inflamed tissues. Once homing to inflamed tissues, these cells gradually downregulate their CCR2 while concomitantly upregulating CX3CR1 as they differentiate into macrophages (41). Interestingly, TAMs exhibit a broad range of CX3CR1 and CCR2 expression levels in a reciprocal pattern (i.e., decreasing CCR2 and increasing CX3CR1), indicating a continuous transformation of these cells from infiltrating monocytes into mature macrophages (42). This dynamic transition of the surface molecules suggests that bone marrow-derived monocytes are highly plastic and that these cells evolve to maturation *in situ* following localization to the tumors (43).

It has been established that bone marrow-derived macrophages and microglia react differently to various types of CNS insults and can perform different functions (44, 45). One example of this from a recent study using a complex parabiosis model showed that peripheral mononuclear cells invade the inflamed CNS during experimental autoimmune encephalomyelitis and play a significant role in disease progression to paralysis (46). By employing GEMMs of PDGFB-driven GBM described above, we have recently shown that the vast majority (up to 85%) of TAMs are infiltrating bone marrow-derived monocytes/macrophages, whereas resident microglia account for the remaining ~15% (42). Bone marrow-derived cells are prominent in perivascular areas, whereas resident microglia is more highly expressed in peritumoral regions (Figure 1B). RNA-sequencing analyses reveal that functional distinctions between bone marrow-derived and microglia-derived TAMs in that genes related to “cellular migration” are mostly enriched in the former, whereas genes associated with “pro-inflammatory cytokines” and “metabolism” are upregulated in the latter (42). These differences may be partially explained by the fact that these two cellular populations arise from distinct progenitors and selectively use different transcription factors for their gene regulation (47). To further illustrate their functional differences, we genetically deleted *Cx3cr1* from the microenvironment of PDGFB glioma-bearing mice and observed an increase in tumor incidence and a shortened survival time of stroma deficient in *Cx3cr1* compared to that in *Cx3cr1* wild-type stroma. These results showed that loss of *Cx3cr1* indirectly promoted trafficking of inflammatory monocytes into the CNS, resulting in a higher accumulation in the perivascular area (17). It did not, however, directly affect the accumulation of microglia in peritumoral regions. The bone marrow-derived monocytes promoted glioma stem-like cells by enhancing their proliferation through the production of IL-1β (17). These data strongly suggest that TAMs derived from the bone marrow compartment drive gliomagenesis, whereas microglia appears to play a less significant role in tumor growth and is mostly involved in tumor cell invasion. Together, these observations lead to several outstanding questions: (a) both human and mouse MES GBM exhibit an increased TAM infiltration when compared to the PN subtype, but do they exhibit different TAM compositions? (b) Is the number of TAMs or their composition more critical in promoting tumor development? (c) How different are TAMs in the CL subtype? Further, does the origin of a TAM matter for its interactions with T cells? These are very important questions that will provide novel insights, which can be used in designing successful immunotherapies aiming at killing tumor cells.

## Immunosuppression in GBM

**Figure 2:**
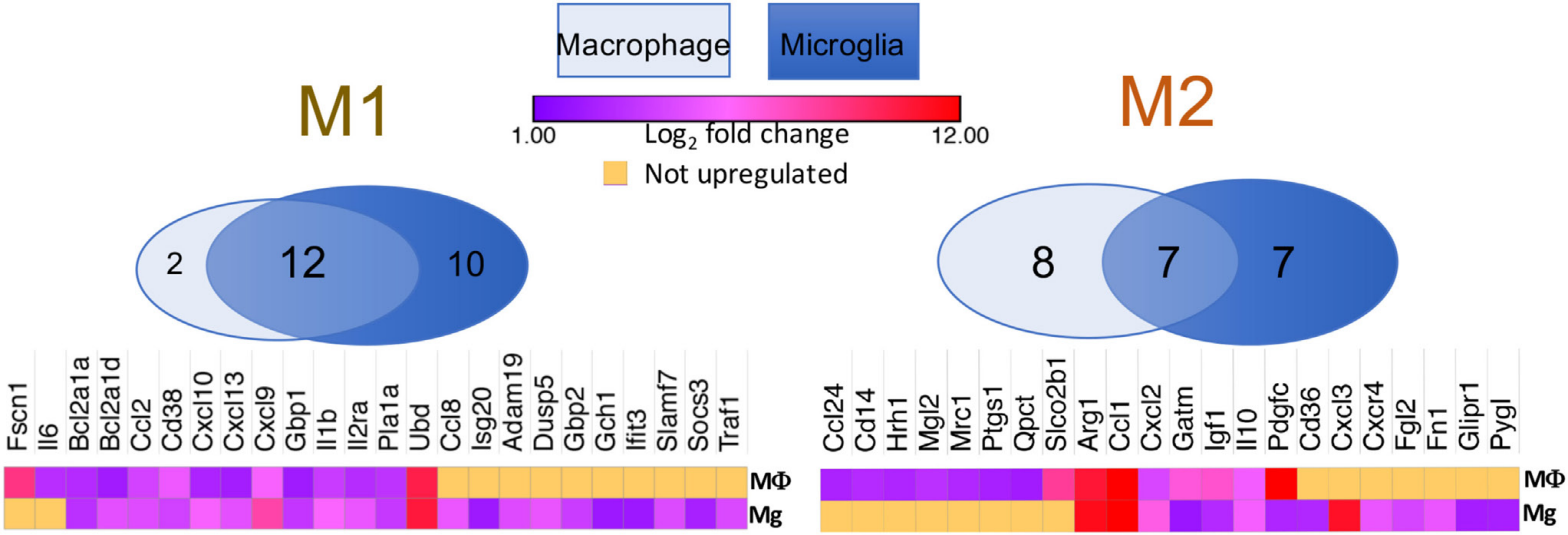
Venn diagrams showing that both M1 and M2 signature genes are present in either BMDM or tumor-associated microglia isolated from a murine model of proneural glioblastoma. Heat maps demonstrate log_2_-fold increases in these genes in BMDM and microglia as compared to their respective naïve controls. Raw RNA-seq data are available at the NCBI Sequence Read Archive database under accession number PRJNA349180 (42).

Tumor-associated macrophages are often considered to be facilitators of tumor growth because of their proangiogenic and immunosuppressive properties. Among these cells are those termed myeloid-derived suppressor cells (MDSCs). Broadly defined, MDSCs in mice are cells that express both CD11b and Gr1 surface markers, and they can be further subdivided into monocytic and granulocytic subtypes. In GBM, the granulocytic MDSCs are rarely found in the tumor (42). The monocytic MDSCs can employ a wide range of mechanisms to suppress cellular immune functions, including upregulation of Arg1 production, induction of T-cell apoptosis, and/or enhancement in the expansion of T_reg_ populations (48). All of these features align with the so-called M2 phenotype. *In vitro* studies initially demonstrated the dichotomous differentiation of macrophages, such that myeloid monocytes can be polarized into classically activated, pro-inflammatory (M1) or alternatively activated, anti-inflammatory (M2) phenotypes (49, 50). M1 cells produce high levels of oxidative metabolites and pro-inflammatory cytokines that are essential for host defense, but can also result in healthy tissue damage (51). On the other hand, M2 cells promote wound healing and suppress adverse immune responses (52). However, despite these initial findings in cell culture experiments, absolute M1 and M2 binary distributions are rare *in vivo*. Subsequently, a range of differentiation has been proposed, with the M1 and M2 phenotypes being at the ends of the spectrum (53). Indeed, in our transcriptome analyses of purified tumor-associated microglia and bone marrow-derived macrophages, we found mixed populations of both M1 and M2 phenotypes in both TAM populations (Figure 2). For instance, the typical M2 marker Arginase1 was upregulated by 10-folds (at log_2_ scale) in both bone marrow-derived macrophages and microglia, whereas IL-1β, a specific M1 cytokine, was also increased by 5-folds in both cell types. However, it is not immediately clear whether these M1 and M2 molecular signatures belong to distinct populations, or if a single cell can express both subsets of molecules at various strengths. What is clear is that TAMs are highly plastic and have been found to switch between M1 and M2 phenotypes in response to their environmental cues (54). Many attempts have been made to polarize TAMs to the M1 fate; however, sustained conversion remains a significant challenge because soluble factors produced by the tumor cells can revert TAMs to an M2 phenotype, despite pharmacological or genetic interventions. A comprehensive understanding of the molecular network that coordinates this conversion will benefit future attempts to maintain a long-lasting antitumor phenotype (55).

## Inhibition or Modulation of TAMs as a Stroma-Directed Strategy

Given that TAMs are elemental accomplices in tumor development, it is reasonable to propose therapeutic options based on inhibiting their infiltration or promoting their demise. MCPs play an essential role in mediating monocyte migration and tissue infiltration. There are four MCP family members in humans—CCL2, CCL7, CCL8, and CCL13, whereas mice express CCL2, CCL7, CCL8, and CCL12. In the setting of murine GBM, we have shown that neoplastic cells in GBM express high levels of CCL2, which contributes to the directional infiltration of CCR2^Hi^ inflammatory monocytes into the tumor (17). When we queried the human TCGA database for CCL2 expression and divided the patients into high and low CCL2 cohorts, we found that GBM patients with a low CCL2 expression survived significantly longer than those with a high CCL2 expression. These findings raise the question as to whether reducing monocyte infiltration by targeting the CCL2–CCR2 axis is a viable option for treating murine PDGFB–GBM, considering that 80% of the TAMs in this subtype are of monocyte origin. To address this question, we showed that genetically interrupting the CCL2–CCR2 axis prolonged the survival of GBM-bearing mice, in agreement with previous pharmacological studies (56, 57). However, in contrast to the promising preclinical studies, neutralizing monoclonal antibodies against CCL2 administered to patients with metastatic, solid tumors did not produce favorable outcomes. Although a similar treatment has not been applied to GBM clinically, caution should be exercised if such an approach is to be considered, because different GBM subtypes maintain different compositions of infiltrating TAMs. Tumors with low levels of bone marrow-derived TAMs may not respond to this therapy. This critical point is also reflected by the fact that anti-VEGFA antibody worked only in the PN subtype when combined with RT, but did not show efficacy in the other GBM subtypes (58).

Microglia relies on colony-stimulating factor 1 (CSF-1) for survival, and CSF-1 receptor inhibitors can effectively eliminate microglia in the brains of naïve mice (35). Although pharmaco-active compounds have demonstrated excellent efficacy in preclinical animal studies against a GEMM of PN GBM, they were not successful in eliminating or decreasing TAM numbers in GBM, suggesting that TAMs gain CSF-1 independence (59). However, a CSF-1 receptor inhibitor failed to provide clinical benefit in non-stratified recurrent GBM patients (60). This failure in translation is likely because TAM heterogeneity was not sufficiently addressed and that there is still a lack of knowledge regarding their differential composition and functions as discussed above. It may also suggest a differential role of CSF-1 in human versus mouse. In order to develop effective therapies, it is paramount that we understand the unique functionalities of TAMs in individual GBM subtypes. RNA-seq analyses of purified populations can provide insights into the pathobiological attributes in tumor development as well as subtype-specific differences.

As discussed above, TAMs are highly plastic and maintain the capability to switch between the tumoricidal M1 and tumorigenic M2 phenotypes. Efforts have been made to achieve “re-education” of TAMs to polarize them toward M1. Nanoparticles, for example, can effectively penetrate solid tumors and locally deliver a drug. Nanoparticles carrying IL-12, which is a Th1-polarizing cytokine, can promote the reversal of TAMs from M2 to M1 (61). To move one step further, it was recently shown that intratumoral delivery of oncolytic virus expressing IL-12 along with systemic administration of anti-CTLA-4 and anti-PD-1 antibodies can significantly prolong the survival of GBM-bearing mice. This beneficial effect was primarily attributed to the M1 polarization of TAMs upon therapy. However, it is interesting to note that the depletion of CD4 T cells can eliminate this therapeutic effect, presenting a previously unappreciated link between TAMs and CD4 T helper cells, as well as tumor death (62).

## Immune Checkpoints and Their Inhibitors in GBM

**Figure 3:**
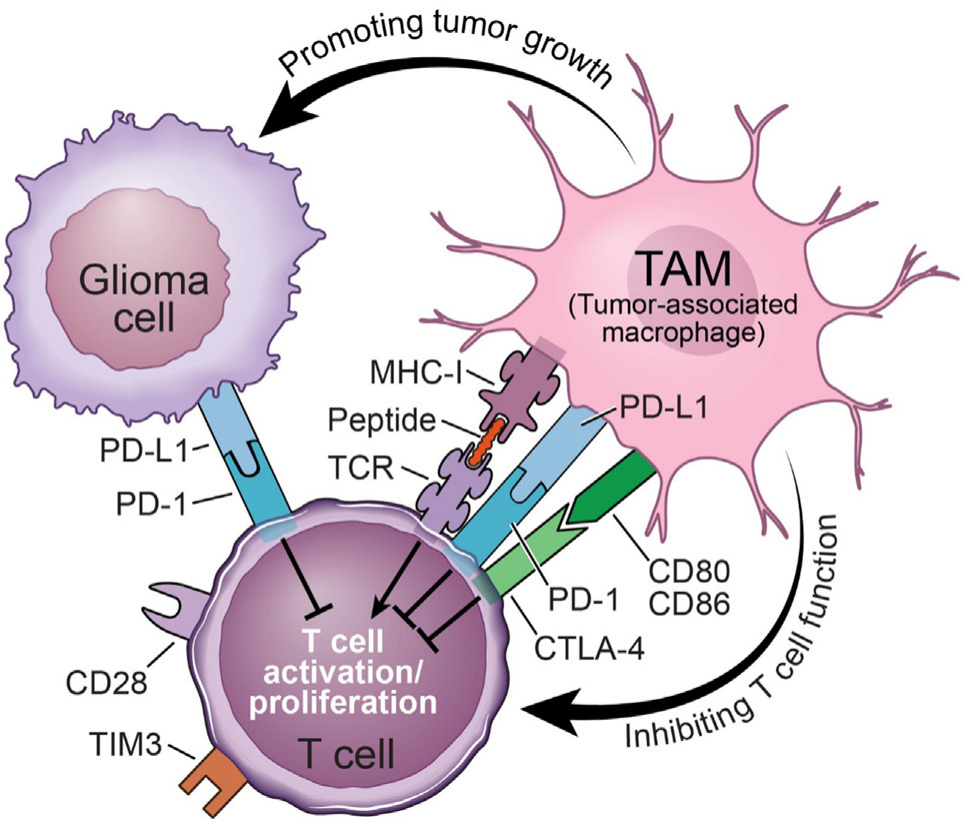
Mechanisms of tumor-associated macrophages (TAMs) inhibiting the functions of tumoricidal T cells in glioblastoma.

Immune checkpoints refer to negative regulatory pathways that function to inhibit T-cell activation and proliferation, thereby maintaining self-tolerance and limiting the duration and amplitude of immune responses (63). Cytotoxic T-lymphocyte-associated antigen-4 (CTLA-4), programmed cell death-1 receptor (PD-1), and T-cell inhibitory receptor (TIM-3) are often found on T cells to perform inhibitory functions through interactions with their corresponding ligands (Figure 3). Studies using PD-1 knockout mice demonstrated that PD-L1 in T cells, antigen-presenting cells (APCs), and host tissue negatively regulated T-cell response (64). Furthermore, in mice, it has been demonstrated that PD-1 is highly expressed by effector T cells during chronic viral infections. By interacting with its ligand PD-L1, which is expressed by stromal cells such as APCs, PD-1 delivers an inhibitory signal to T cells to attenuate their proliferation and effector functions, which can be reversed by using PD-L1-neutralizing antibodies (65). The presence of PD-1 on the surface of these T cells also serves as an indicator of functional exhaustion (65). These and many other elegant discoveries regarding immune checkpoint inhibitors have entered the field of oncology [for a detailed description, see Ref. (66)]. Tumors have evolved to abduct this system for their own benefit by co-opting the cells in the microenvironment, e.g., TAMs, to express high levels of PD-L1. It was recently documented that both the number of PD-1^+^ tumor-infiltrating lymphocytes and PD-L1 expression are significantly increased in GBM, providing a rationale for the use of immune checkpoint blockade to interrupt the PD-1/PD-L1 axis as a potential therapy for GBM (67–69). Even though the data on the expression of PD-1/PD-L1 in GBM patients are largely correlative based on immunohistochemical antibody staining or TCGA data mining (67, 70, 71), they nevertheless represent the first steps forward in a new area of research in the GBM field, which is to understand the biological function of PD-1/PD-L1, T-cell infiltration and function, and their interaction with TAMs.

Immune checkpoint inhibitors such as anti-CTLA-4 antibody (Ipilimumab) and anti-PD-1 antibodies (Nivolumab or Pembrolizumab) were approved by the FDA for the treatment of non-resectable or metastatic melanoma and have been successful. However, monotherapy with anti-PD-1 or combinational therapy with anti-PD-1 and anti-CTLA-4 antibodies for the treatment of recurrent GBM recently failed in phase III clinical trial (72). This failure in translation implies that the simple blockade of immune checkpoints may not restore the tumoricidal functions of T cells, which may be intrinsically impaired or exhausted. For instance, it has been found that PD-1 expression on CD4 cells identifies a dysfunctional subset refractory to rescue with PD-1 blockade, suggesting that the influence of immune checkpoint inhibitors may involve the recovery of function in the PD-1–CD4^+^ T-cell compartment (73). It may also imply yet again that GBM should not be viewed as a single entity, but rather a complex tumor of molecular subtypes, which may not respond equally to a given therapy. Indeed, about 8% of all patients in this trial responded well to Nivolumab (72). Although their molecular subtypes have not been characterized, it is possible that these patients maintain a molecular commonality that is sensitive to this therapy. In addition, the functional significance of PD-1/PD-L1 blockade should be evaluated beyond correlative studies.

TIM-3 is enriched in GBM and IDH-wild-type gliomas. TIM-3 is a protein encoded by the *HAVCR2* gene that mediates T-cell-mediated immune functions such as the response to tumor cells and cytotoxicity directed against tumor cell targets. It also mediates similar inflammatory activation functions as PD-L1 in glioma. Interestingly, TIM-3 is a potential marker for the MES molecular subtype. Clinically, the high expression of TIM-3 has been shown to be an independent indicator of poor prognosis. All of these factors make TIM-3 a potential focal point for immunotherapeutic strategies when gliomas gain resistance to antibodies against PD-1/PD-L1 (74).

## Concluding Remarks

Recent advances in cancer immunotherapy have created great enthusiasm and anticipation for an effective treatment for GBM. Most of the current cancer immune therapies, however, focus on the importance of cytotoxic T cells. This may undervalue the significance of innate immune components in the tumor microenvironment, such as TAMs. Tumors are highly adaptive and maintain abundant non-neoplastic cells; therefore, concomitant therapies involving multiple aspects that simultaneously target tumor cells, TAMs, and T cells should be considered. In this regard, it has been shown that blocking TAM-mediated immunosuppression holds great promise for increasing the efficacy of gene therapy-mediated immunotherapies for GBM (75). Further, considering the robust differences in molecular signaling, TAM composition, and T-cell abundance between GBM subtypes, combinatorial therapy with subtype-specific considerations could yield greater success for future GBM immunotherapies.

## Author Contributions

Both ZC and DH contributed to conceive the article, review the literature, and write the manuscript.

## Conflict of Interest Statement

The authors declare that the research was conducted in the absence of any commercial or financial relationships that could be construed as a potential conflict of interest.
